# System Analysis Identifies MYBL2 As a Novel Oncogene Target for Metastatic Prostate Cancer

**DOI:** 10.7150/jca.107232

**Published:** 2025-02-11

**Authors:** Renlun Huang, Jing Li, Jiawei Zhu, Wei Deng, Zhichao Wang, Songtao Xiang

**Affiliations:** 1 The Second Clinical College of Guangzhou University of Chinese Medicine, Guangzhou, Guangdong, China.; 2 Department of Urology, Guangdong Provincial Hospital of Chinese Medicine, Guangzhou, Guangdong, China.; 3 Guangdong Provincial Key Laboratory of Clinical Research on Traditional Chinese Medicine Syndrome, Guangdong Provincial Academy of Chinese Medical Sciences, Guangzhou, China.

**Keywords:** MYBL2, NOTCH3, prostate cancer, EMT, bone metastasis

## Abstract

Bone metastasis significantly contributes to the unfavorable prognosis observed in patients with prostate cancer. MYB proto-oncogene like 2 (MYBL2) has been identified as a potential target gene implicated in tumor progression. Nevertheless, the oncogenic role and underlying mechanisms of MYBL2 in bone metastasis of prostate cancer (PCa) have yet to be elucidated. Bioinformatics analyses were employed to identify genes pivotal to metastatic PCa. Subsequently, a series of molecular biology experiments in vitro, alongside a model of PCa bone metastasis in vivo, were utilized to validate the pro-metastatic effects and underlying mechanisms of MYBL2. A bioinformatics analysis identified a candidate set of 72 genes, which was used to establish a PFS prognostic model highlighting 16 key genes. Based on the expression of these 16 key genes, 498 patients with PCa from the TCGA database were divided into four subgroups. Patients in the C1 and C4 subgroups had poorer prognoses. Through the analysis of sequencing data from the C1 and C4 cohorts in comparison to the C2 and C3 cohorts, we identified MYBL2 as a critical prognostic gene in metastatic PCa. Notably, we found that MYBL2 was significantly expressed in metastatic PCa and positively related to poor prognosis. Mechanistic studies revealed that MYBL2 overexpression promoted PCa cells invasion and EMT, while NOTCH3 knockdown partly abrogated that. Moreover, MYBL2 overexpression can promote PCa xenograft growth and bone metastasis in vivo. This study found that MYBL2 overexpression in PCa were positively related to metastasis and poor prognosis. MYBL2 promoted PCa bone metastasis via activating NOTCH3. Targeting the MYBL2/NOTCH3 axis could help prevent metastatic PCa.

## Introduction

Prostate cancer (PCa) is the second most prevalent malignancy among men and is the fifth most frequently diagnosed cancer globally 1. Unfortunately, most patients with PCa are diagnosed in the middle to late stages, and effective treatment options are limited. Metastasis primarily leads to poor prognosis in patients with PCa. Bone is involved in 70% of metastatic cases, and the five-year survival rate for patients diagnosed with metastatic PCa is below 30% 2. Therefore, a comprehensive understanding of the potential mechanisms of metastatic PCa is essential.

MYB proto-oncogene like 2 (MYBL2), a transcription factor belonging to the MYB family, plays a crucial role in regulating the cancer cell cycle progression, cellular survival, and differentiation 3. Notably, MYBL2 is commonly overexpressed in various solid tumors and is significantly associated with poor prognosis 4. Specifically, silencing of MYBL2 expression increased the sensitivity of hormone-sensitive PCa cells to androgen deprivation and taxane-based therapies in vitro 5. Moreover, the increased expression of MYBL2 was positively associated with the advanced* TNM* stage, high PSA level, and advanced Gleason score in patients with PCa 6. Another study suggested that MYBL2 was a novel independent prognostic biomarker 7. Further investigation is warranted to elucidate the oncogenic role and underlying mechanisms of MYBL2 in metastatic PCa.

In this study, we identified MYBL2 as a significant responsive gene for poor prognosis in patients with PCa. Furthermore, MYBL2 overexpression not only enhanced PCa cells invasion and EMT, but also promoted PCa xenograft growth and bone metastasis in vivo. Mechanistic studies revealed that MYBL2 overexpression significantly boosted PCa invasion by increasing the expression of NOTCH3. This study identified MYBL2/NOTCH3 axis as a new target for preventing PCa metastasis.

## Materials and methods

### Data retrieval and analysis

These meaningful data were sourced from* the Cancer Genome Atlas* (TCGA) database (https://tcga-data.nci.nih.gov/tcga/) and* Gene Expression Omnibus* (GEO) database (http://www.ncbi.nlm.nih.gov/geo). High-throughput sequencing and clinical data from 498 prostate cancer patients in the TCGA database **(S1 Table, S2 Table)** were analyzed using R software (version 4.0.3, R foundation for statistical computing, 2020) to identify main genes linked to poor prognosis of patients with prostate cancer.

### Venn diagrams

The correlation among various sequencing datasets of metastatic prostate cancer was analyzed using Venn diagrams, which were generated through a web-based tool (http://www.bioinformatics.com.cn/static/others/jvenn/example.html) 8.

### Consensus clustering analysis

High-throughput sequencing data were sourced from TCGA database **(S1 Table)**, accompanied by clinical information pertaining to 498 prostate cancer patients **(S2 Table)**. For consensus clustering analysis, the *ConsensusClusterPlus* R package (version 1.54.0) was utilized. The cluster heatmap was generated and analyzed using the *Pheatmap* R package (version 1.0.12), and the top 25% of genes were selected for visualization. All aforementioned analytical procedures were executed using R software (version 4.0.3).

### Immune infiltration

Immune infiltration analysis in prostate cancer was performed using STAR-counts data and clinical information from the TCGA database. The *Immunedeconv* R package, incorporating six algorithms (CIBERSORT, MCP-counter, and EPIC), was used for this analysis in R software (version 4.0.3).

### Pan-cancer analysis

RNA-Seq level_3_ expression data and corresponding clinical information for 10,228 patients across 32 tumor types were downloaded from TCGA database. Data obtained from the GTEx database were used as normal controls 9. These data were subsequently processed utilizing the R package *ggplot2* implemented by R software (version 4.0.3).

### Cell culture

The PC3, and DU145 cell lines were obtained from *American Type Culture Collection* (ATCC, MD, USA) and cultured in 1640 or DMEM Medium (Gibco, Grand Island, NY, USA) with 1% penicillin-streptomycin and 10% fetal bovine serum (Gibco). These cells must be cultured at 37 °C with 5% CO_2_.

### Plasmid transfection

Recombinant NOTCH3 shRNA plasmids and MYBL2 overexpression plasmids, along with a non-target control plasmid, were obtained from *Vigene Biosciences* (WZ Biosciences Inc., China). The experiments were conducted in accordance with the protocols specified by the reagent manufacturer.

### Transwell assay

A transwell assay were performed to assess changes in the invasion abilities of DU145 and PC3 cells. The basement membrane of the upper chamber was coated with Matrigengel (082703, ABW^®^, Shanghai, China).

### Western blotting

Western blotting was performed following the manufacturer's guidelines, using Vimentin (60330-1-Ig, Proteintech), E-cadherin (60335-1-Ig, Proteintech), β-actin Rabbit (8457S, CST), and β-actin Mouse (3700S, CST).

### QPCR

A quantitative PCR (qPCR) assay was employed to assess alterations in NOTCH3 mRNA expression following MYBL2 overexpression. Initially, total cellular RNA was extracted utilizing the SteadyPure Universal RNA Extraction Kit (AG21017, AG, Accurate Biotechnology (Hunan) Co., Ltd, Hunan, China). Subsequently, Genomic DNA was removed and the total RNA was reverse transcribed into complementary DNA (cDNA) with the *Evo M-MLV* RT Mix Kit with gDNA Clean for QPCR kit (AG11728, AG). The complementary DNA (cDNA) was amplified utilizing the SYBR® Green Premix Pro Taq HS qPCR Kit (ROX Plus) (AG11718, AG) and was then conducted on the ABI QuantStudio 7 Flex Real-Time PCR System (Applied Biosystems). Target gene expression levels were measured using the 2^(-ΔΔCT) method. The primer sequences of NOTCH3 were REVERSE: GGTTCAAGCGATTCTCCTTCCTCAG; FORWARD: CATGGTGCCAGTTGCCTGTAGTC.

### RNA sequencing analysis

To investigate alterations in gene expression in PC3 cells following the overexpression of MYBL2, high-throughput RNA sequencing (RNA-Seq) was performed by Genedenovo Biotech Co., Ltd. (Guangzhou, China) utilizing the HiSeqTM 4000 sequencing platform (Illumina, California, USA). Gene abundance was quantified and normalized using Reads Per Kilobase of transcript per Million mapped reads (RPKM). The edgeR package (available at http://www.r-project.org/) was employed to identify differentially expressed genes (DEGs) between the MYBL2 overexpression and control groups. The threshold for significant fold change in target genes was established as |log2FC| > 0.58 with *p* value < 0.05. The raw RNA sequencing data have been uploaded to **S3 Table**.

### Immunohistochemistry (IHC) assay and hematoxylin-eosin staining (HE) assay

The expression of target proteins was detected using a universal two-step method detection kit (PV-9000, ZSGB-BIO, Beijing, China). Tissue sections should be deparaffinized, followed by antigen repair and blocking endogenous peroxidase. Subsequently, the sections are incubated with a primary antibody, and color development is achieved through DAB staining and hematoxylin (C0105M, Beyotime). An immune response score (IRS) method was used to semi-quantify the expression of target proteins 10. The Immune Response Score (IRS) was calculated as the product of the positive cell score and the staining intensity score.

Hematoxylin-eosin staining was performed to analyze the cellular structure of the tissue according to the instructions provided by the manufacturer. Bone tissue needs to be decalcified using EDTA decalcification solution (AR1071, BOSTER, Hubei, China). Similarly, after in turn dehydration, embedding, sectioning and dewaxed, bone tissue can be stained with 10% hematoxylin solution (C0105M, Beyotime) for nucleus and 1% eosin solution (C0105M, Beyotime) for cytoplasm.

### Immunofluorescence (IF) assay

First, the tumor tissues were embedded in optimal cutting temperature compound (OCT, 4583, SAKURA). Subsequently, the tissues were frozen and sectioned on low-temperature environment. Tissue sections were initially incubated with primary antibodies, followed by a fluorescence incubation with secondary antibodies. Subsequently, the nuclei were stained using DAPI (P0131, Beyotime). Fluorescence imaging was conducted utilizing an LSM710 confocal microscope (Zeiss, Jena, Germany). The following antibodies were using: MYBL2 (ab314862, abcam, Cambridge, UK), NOTCH3 (ab23426, abcam), and anti-rabbit IgG-Alexa Fluor® 555 (8953S, CST).

### Animal experiment

Four-week-old male C57BL/6 mice were procured from certified suppliers and subsequently housed and experimentalized in the Specific Pathogen-Free (SPF) animal facility at the Laboratory Animal Center of Guangdong Provincial Hospital of Traditional Chinese Medicine, which was approved by the Animal Experiment Ethics Committee of Guangdong Provincial Hospital of Traditional Chinese Medicine (approval No. 2018071). A mice bone metastasis model was employed to investigate the effect of MYBL2 overexpression on prostate cancer xenograft. Tumor volumes (V) were calculated using the method: V = (length × width^2)/2. In vivo imaging was conducted using the small animal imaging system. Bone metastasis and bone degradation were identified and assessed utilizing a micro-computed tomography (micro-CT) scanning system (SKYSCAN 1276, BRUKER). Isoflurane (R510-22-10, RWD, China) served as the gaseous anesthetic agent in the animal studies.

### Statistical analyses

The study's statistical analyses were performed with SPSS 24.0 (Abbott Laboratories, Chicago, USA). Results are presented as mean ± SD, and *p* values less than 0.05 considered as statistically significance.

## Results

### Differentially expressed genes were identified between metastatic and non-metastatic prostate cancer tissues as well as related to poor prognosis

**Figure 1:**
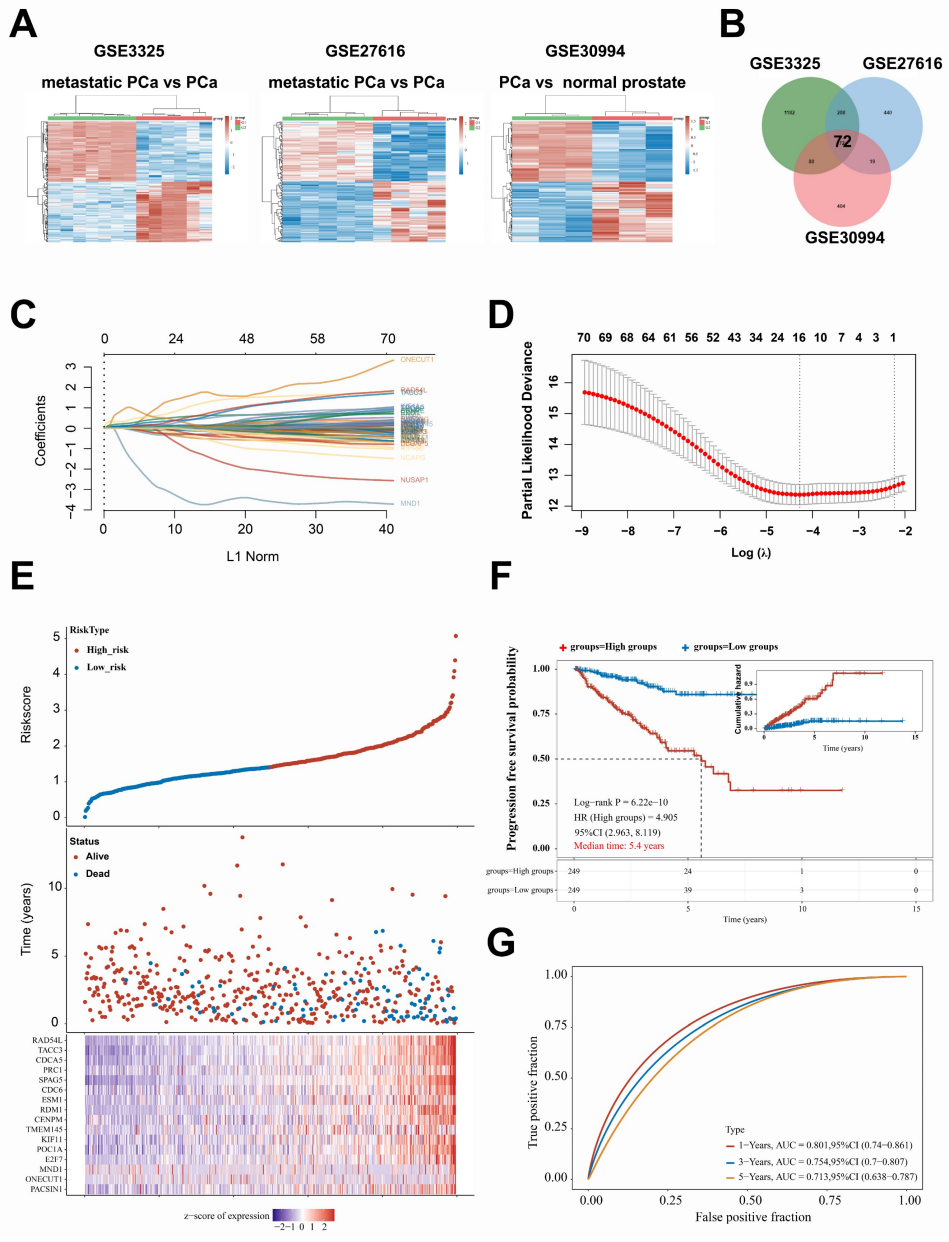
** Differentially expressed genes were identified between metastatic and non-metastatic prostate cancer tissues as well as related to poor prognosis. A**. Heat map, the differential gene expression in GSE3325, GSE27616, and GSE30994, G1: metastatic PCa or primary PCa; G2: primary PCa or normal prostate. **B**. Venn diagram, the intersection gene set of differential gene expression among in GSE3325, GSE27616, and GSE30994. **C**. The prediction model was structured based on 72 differentially expressed genes and PFS of patients with PCa; the horizontal axis (abscissa) denotes the values of the independent variable, denoted as lambda, while the vertical axis (ordinate) illustrates the corresponding coefficients of this independent variable. **D**. The relationship between partial likelihood bias and log (λ) was analyzed using the LASSO Cox regression model.** E**. The uppermost graph illustrates the scatter distribution of high-risk and low-risk cohorts as identified by the model, with red denoting the high-risk cohort and blue indicating the low-risk cohort. The median scatter plot depicts the distribution of survival time and survival status in relation to gene expression across various samples. The subsequent figure presents a heat map of gene expression incorporated within the model. **F**. progression-free survival (PFS) analysis of high-risk and low-risk cohorts. **G**. The AUC-ROC curve of the PFS prediction model was employed to assess the predictive accuracy for 1-, 3-, and 5-year survival rates among patients with prostate cancer. CI, confidence interval; HR, Hazard Ratio; AUC, Area Under the Curve.

An intersection analysis was performed to identify highly expressed genes associated with metastatic prostate cancer (PCa) utilizing the *Gene Expression Omnibus* (GEO) dataset GSE3325, GSE27616, and GSE30994** (Fig. 1A)**. Seventy-two candidate genes exhibiting differential expression in metastatic PCa tissues were identified and analyzed** (Fig. 1B)**. More importantly, we obtained STAR-counts data and clinical information from the TCGA database to create a PFS prediction model for PCa patients using the expression data of 72 candidate genes. Subsequently, we identified the genes that significantly influence the PFS of patients with PCa. The prediction model was as follow: Riskscore = (0.1495) × PACSIN1 + (0.6936) × ONECUT1 + (-2.4826) × MND1 + (-0.0887) × E2F7 + (0.0261) × POC1A + (-0.253) × KIF11 + (0.0418) × TMEM145 + (-0.1534) × CENPM + (0.4033) × RDM1 + (0.0868) × ESM1 + (-0.1711) × CDC6 + (0.5707) × KIF11 + (0.0418) × TMEM145 + (-0.1534) × CENPM + (0.4033) × RDM1 + (0.0868) × ESM1 + (-0.1711) × CDC6 + (0.5707) × SPAG5 + (0.6354) × PRC1 + (0.0433) × CDCA5 + (0.281) × TACC3 + (0.1666) × RAD54L **(Fig. 1C-E)**. The results indicated that the prediction model functions as a risk model** (**HR = 4.905,** Fig. 1F)**. Furthermore, the prognosis for patients classified in the high-risk group by this model was significantly poorer compared to those in the low-risk group, with a median progression-free survival time of 5.4 years (*p* = 6.22e-10)** (Fig. 1F)**. Furthermore, the model demonstrated robust performance in predicting progression-free survival at 1, 3, and 5 years (AUC exceeding 0.7, **Fig. 1G**). In conclusion, this study identified the key genes involved in PCa metastasis by bioinformatics analysis.

### A set of 498 samples data from TCGA were stratified and analyzed based on the genes included in the prediction model

**Figure 2:**
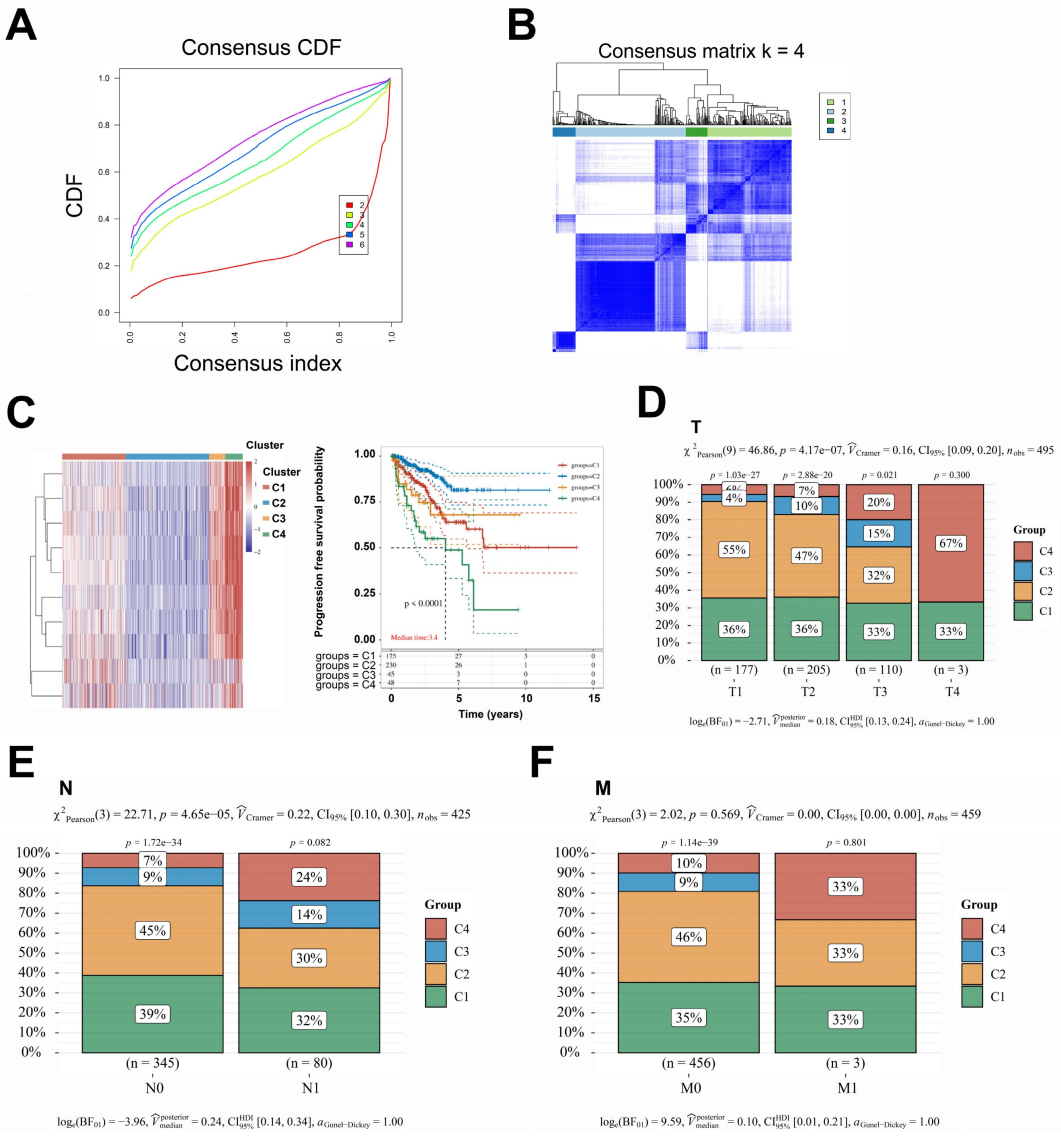
** A set of 498 samples data from TCGA were stratified and analyzed based on the genes included in the prediction model. A**. Cumulative distribution function (CDF) curves for subtype classification; Heatmap of consistency clustering results, with different colors representing different subtypes. **B**. Heatmap, the top 25% of genes were selected for visualization among C1, C2, C3, and C4 groups. **C**. The PFS survival curves of C1, C2, C3, and C4 groups. **D-F**. Comparison of *TNM* staging between C1, C2, C3, and C4 groups.

**Figure 3:**
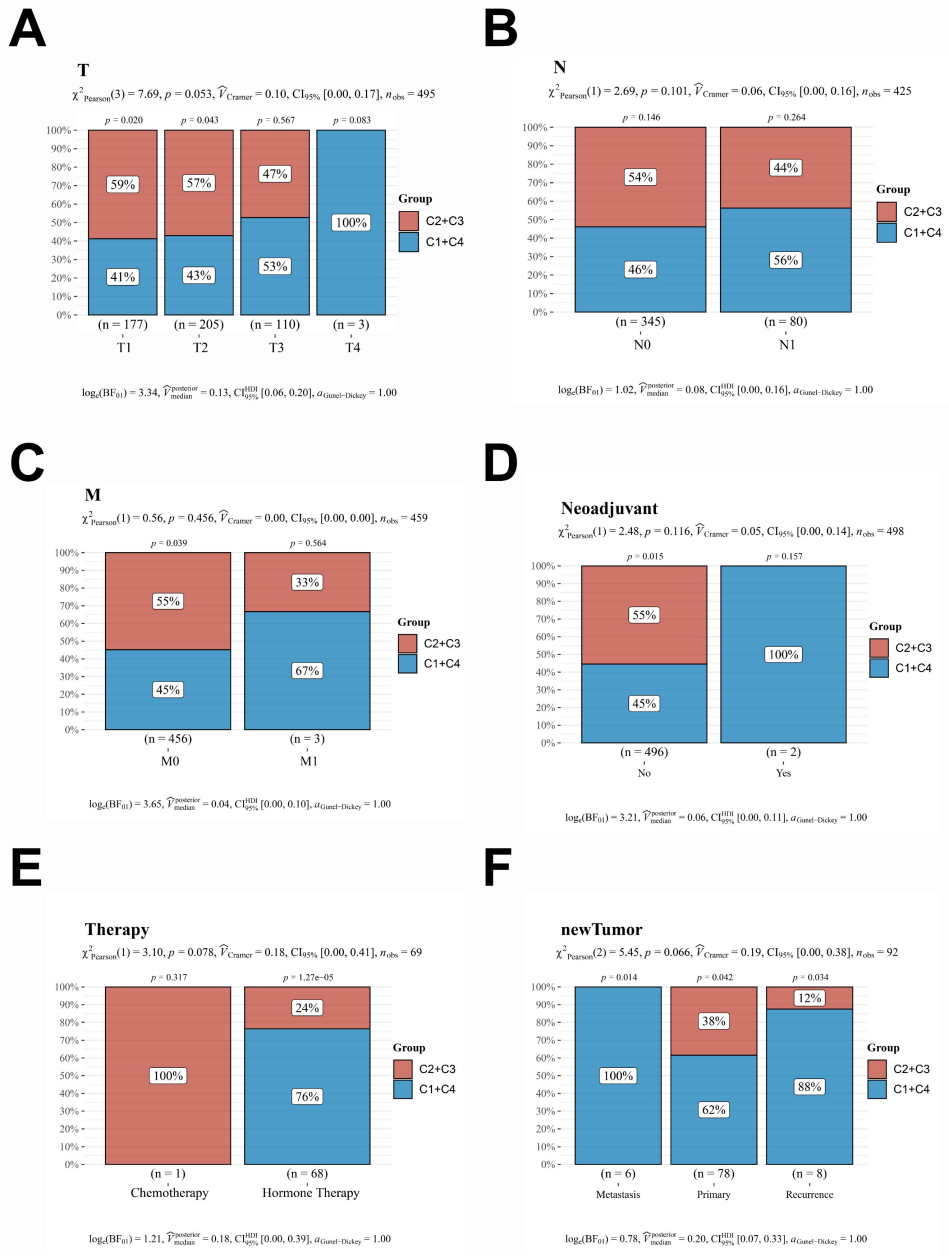
** A comprehensive reanalysis of patient data across the four distinct groups. A-C**. The data from groups C1 and C4, which exhibited a poorer prognosis; conversely, the data from groups C2 and C3, which demonstrated a more favorable prognosis. **D-F**. The treatment strategies and prognostic outcomes between the groups C1 + C4 and C2 + C3 were systematically compared. CDF, Cumulative Distribution Function; CI, confidence interval.

To further investigate the correlation between this prediction model and the prognosis of patients with PCa, the dataset of 498 patients selected from the TCGA database was classified according to the expression levels of the 16 genes included in the model: *PACSIN1, ONECUT1, MND1, E2F7, POC1A, KIF11, TMEM145, CENPM, RDM1, ESM1, CDC6, SPAG5, PRC1, CDCA5, TACC3,* and* RAD54L*. These 498 patients were classified into four groups (C1, C2, C3, and C4) **(Fig. 2A-B)**. Notably, patients in the C1 and C4 groups exhibited worse progression-free survival (PFS) and higher *TNM* stages** (Fig. 2C-F)**. However, no significant differences in* N* stages and *M* stages were observed among the four groups **(Fig. 2E-F)**. To enhance the accuracy of identifying patients with higher malignancy in PCa, we amalgamated the data from groups C1 and C4, as well as from groups C2 and C3. Significantly, the comparative analysis of TNM staging between groups C1 + C4 and C2 + C3 demonstrated that group C1 + C4 exhibited a markedly higher-grade *TNM* stage** (Fig. 3A-C)**. Despite undergoing more comprehensive treatment, patients in group C1 + C4 also demonstrated a higher incidence of metastasis and recurrence **(Fig. 3D-F)**. In conclusion, this study effectively identified cohorts of patients demonstrating advanced clinicopathological features of PCa.

### MYBL2 expression was positively correlation with poor prognosis and macrophages infiltration in prostate cancer

**Figure 4:**
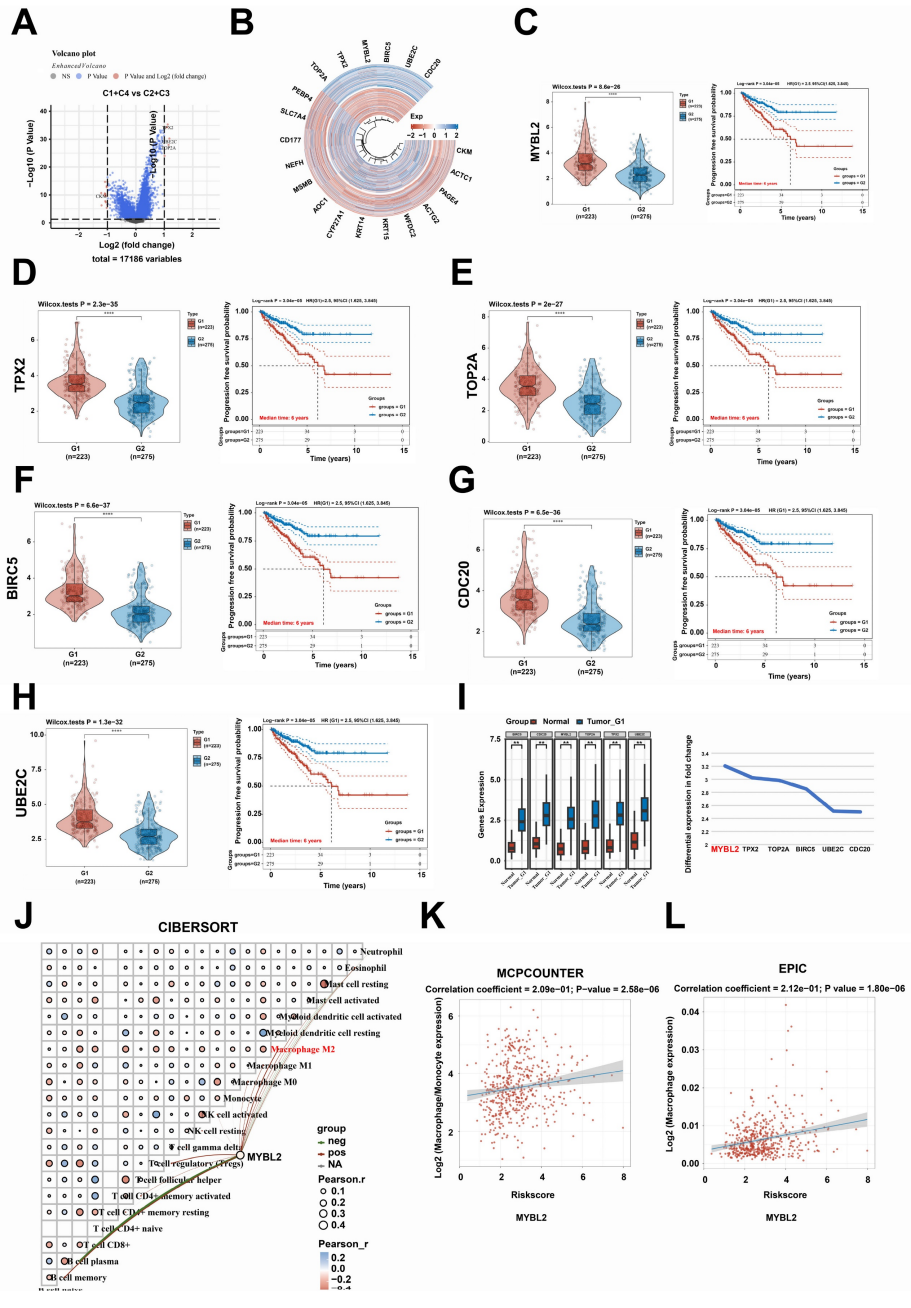
** MYBL2 expression was positively correlation with poor prognosis and macrophages infiltration in prostate cancer. A**. Volcano plot, Comparison of differentially expressed genes between groups C1 + C4 and C2 + C3. **B**. Heatmap, Comparison of differentially expressed genes between groups C1 + C4 and C2 + C3. **C-H**. The relationship between *TOP2A, TPX2, MYBL2, BIRC5, UBE2C,* and* CDC20* expression and PFS survival of patients with prostate cancer. **I**. Differential expression of *TOP2A, TPX2, MYBL2, BIRC5, UBE2C,* and *CDC20* between prostate cancer tissue and normal prostate tissue. And the fold change ranking of *TOP2A, TPX2, MYBL2, BIRC5, UBE2C,* and *CDC20* was analyzed in prostate cancer compared to normal prostate tissue. **J-L**. The relationship between the expression levels of *MYBL2* and the infiltration of various immune cell types. **, *p* < 0.01; ****, *p* < 0.0001. CI, confidence interval; HR, Hazard Ratio, SE, standard error.

We conducted an analysis of differentially expressed genes between the groups C1 + C4 and C2 + C3 **(Fig. 4A-B)**. Our findings indicated that exhibited significantly elevated expression levels in the group C1 + C4, which were strongly associated with poor prognosis in patients with prostate cancer (PCa) **(Fig. 4C-H)**. More importantly, our findings indicated that the differential expression of MYBL2 was most pronounced in PCa tissues **(Fig. 4I)**. Moreover, MYBL2 demonstrates a significant correlation with immune cell infiltration within the tumor microenvironment (TME) of PCa, with a specific positive association observed with macrophage infiltration** (Fig. 4J-L)**. In conclusion, the expression of MYBL2 was positively associated with the infiltration of macrophage and was significantly correlated with poor prognosis in patients with PCa.

### Pan-cancer analysis revealed MYBL2 as an oncogene across multiple tumor types

**Figure 5:**
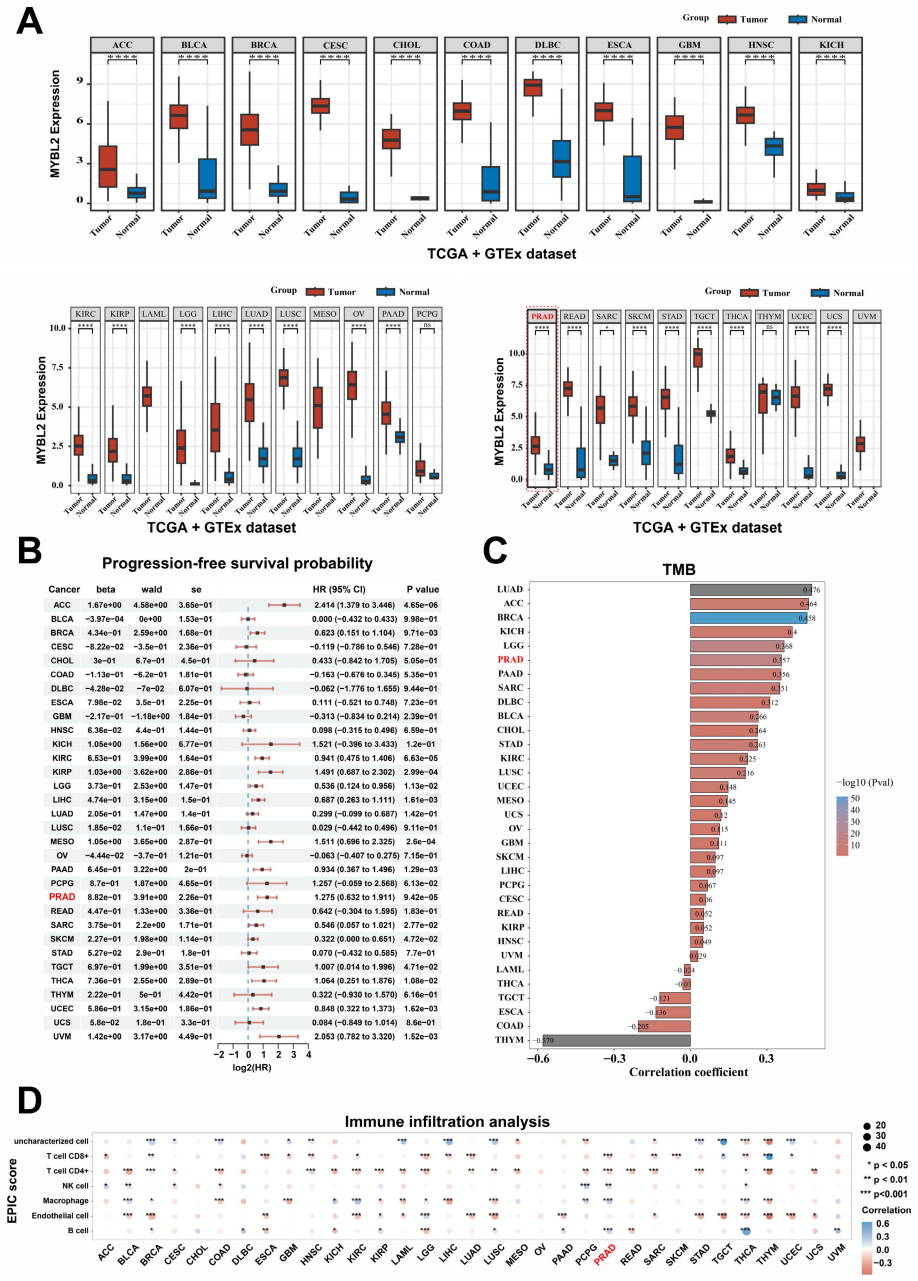
** Pan-cancer analysis of MYBL2 across multiple tumor types. A**. Comparison of differential expression of MYBL2 between tumor and normal in 32 tumors types. **B**. MYBL2 expression and PFS survival of patients with 32 tumors types. **C**. Spearman correlation analysis between TMB and MYBL2 gene expression is depicted. **D**. The relationship between the expression levels of *MYBL2* and the infiltration of various immune cell types was assessed using the EPIC algorithm. *, *p* < 0.05; **, *p* < 0.01; ***, *p* < 0.001. GTEx, The Genotype-Tissue Expression. HR, Hazard Ratio; Pval, *p* value; TMB, tumor mutational burden; ACC, adrenocortical carcinoma; BLCA, bladder urothelial carcinoma; BRCA, breast invasive carcinoma; CESC, cervical squamous cell carcinoma and endocervical adenocarcinoma; CHOL, cholangiocarcinoma; COAD, colon adenocarcinoma; DLBC, lymphoid neoplasm diffuse large B-cell lymphoma; ESCA, esophageal carcinoma; GBM, glioblastoma multiforme; HNSC, head and neck squamous cell carcinoma; KICH, kidney chromophobe; KIRC, kidney renal clear cell carcinoma; KIRP, kidney renal papillary cell carcinoma; LGG, brain lower grade glioma; LIHC, liver hepatocellular carcinoma; LUAD, lung adenocarcinoma; LUSC, lung squamous cell carcinoma; MESO, mesothelioma; OV, ovarian serous cystadenocarcinoma; PAAD, pancreatic adenocarcinoma; PCPG, pheochromocytoma and paraganglioma; PRAD, prostate adenocarcinoma; READ, rectum adenocarcinoma; SARC, sarcoma; SKCM, skin cutaneous melanoma; STAD, stomach adenocarcinoma; TGCT, testicular germ cell tumors; THCA, thyroid carcinoma; THYM, thymoma; UCEC, uterine corpus endometrial carcinoma; UCS, uterine carcinosarcoma; UVM, uveal melanoma.

In this study, utilizing the TCGA and GTEx datasets for a comprehensive pan-cancer analysis, we observed that MYBL2 was overexpressed in nearly all tumor types, including ACC, BLCA, CESC, BRCA, COAD, CHOL, DLBC, GBM, ESCA, HNSC, KIRC, KICH, KIRP, LIHC, LGG, LUSC, LUAD, PAAD, OV, PRAD, SARC, READ, STAD, SKCM, THCA, TGCT, UCEC, and UCS **(Fig. 5A)**. Furthermore, the overexpression of MYBL2 was associated with poor prognosis across various tumor types including ACC, KIRC, BRCA, LGG, KIRP, PAAD, LIHC, THCA, PRAD, and UCEC **(Fig. 5B)**. More significantly, our findings indicated a positive correlation between MYBL2 expression and tumor mutational burden (TMB), particularly in prostate adenocarcinoma (PRAD) **(Fig. 5C)**. This observation suggested that MYBL2 may serve as a potential biomarker for predicting the efficacy of immunotherapy in patients with prostate adenocarcinoma (PRAD). Consequently, we extended our investigation to assess MYBL2 expression across various tumor types and its association with immune infiltration. Our analysis revealed that MYBL2 expression was correlated with immune cell infiltration in multiple tumor contexts **(Fig. 5D)**. In summary, this study demonstrated that MYBL2 was markedly overexpressed across various tumor types and was positively related to poor prognosis of patients with cancer. Furthermore, MYBL2 served as a predictive marker for increased tumor mutational burden (TMB) in patients with cancer, with this effect being particularly pronounced in prostate adenocarcinoma (PRAD).

### MYBL2 overexpression promotes the invasion and EMT of prostate cancer cells via increasing NOTCH3 expression

**Figure 6:**
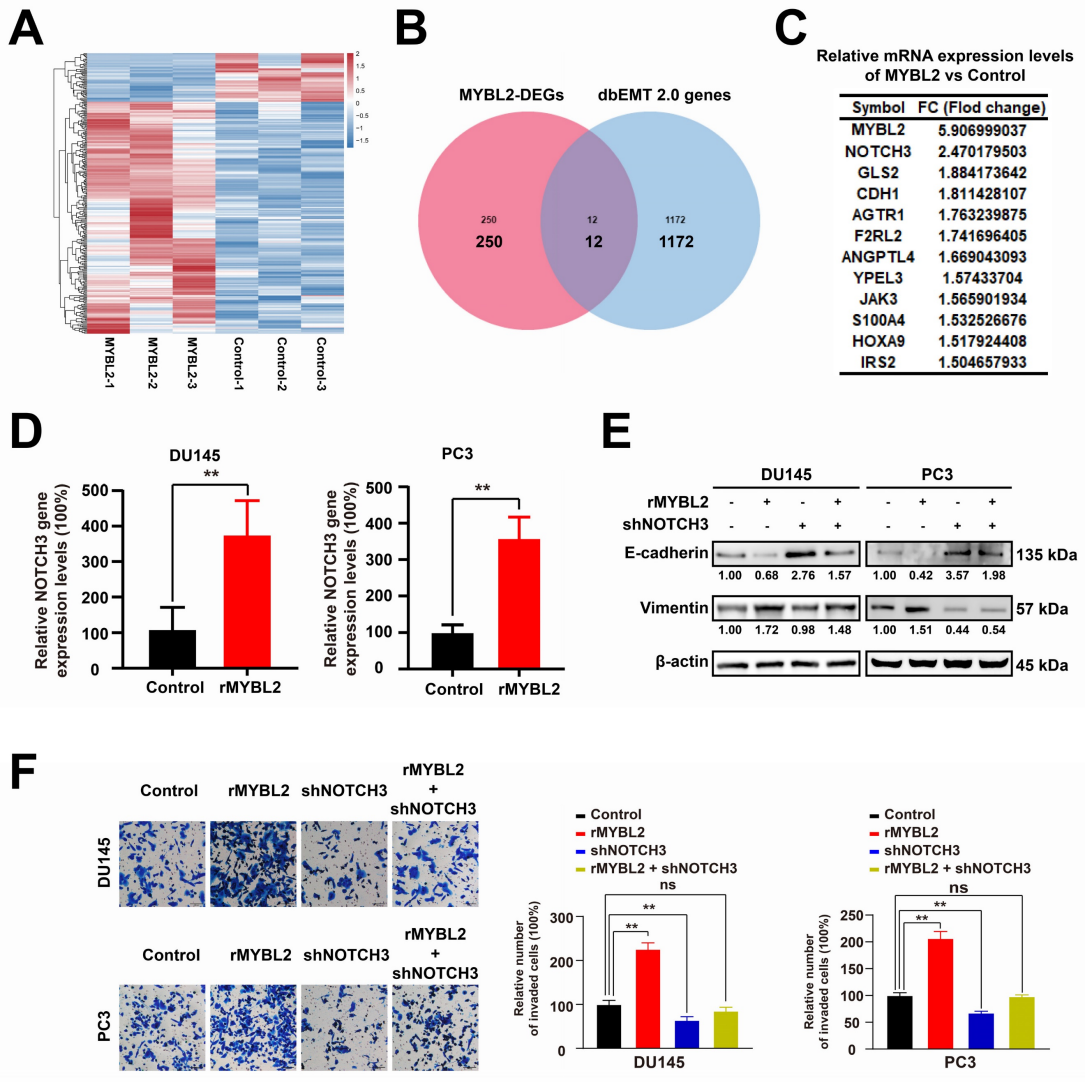
** MYBL2 overexpression promotes the invasion and EMT of prostate cancer cells via increasing NOTCH3 expression. A**. Heatmap, genes differentially expressed in PC3 cells following MYBL2 overexpression. **B**. Venn diagrams; 1184 EMT-related genes were obtained from dbEMT 2.0 database (http://dbemt.bioinfo-minzhao.org/index.html). **C**. A gene cluster containing twelve responsive genes. **D**. *MYBL2* overexpression promoted the *NOTCH3* expression. **E-F**. MYBL2 overexpression promoted invasion and EMT of prostate cancer cells, while the CDH1 NOTCH3 knockdown partly abrogated that. All values are presented as the mean ± SD. n = 3, **p < 0.01; ns represents no statistical significance. DEGs, differentially expressed genes; rMYBL2, the recombinant overexpression plasmid of MYBL2; shNOTCH3, the recombinant shRNA plasmid of NOTCH3.

Elucidating the oncogenic role of MYBL2 may unveil potential therapeutic targets for metastatic prostate cancer (PCa). Consequently, we conducted further investigations to determine whether modulating MYBL2 expression influences PCa metastasis. In this study, RNA-seq analysis was employed to elucidate the alterations in gene expression associated with MYBL2 overexpression in PC3 cells, and 262 differentially expressed genes were identified (|log2FC| > 0.58, *p* < 0.05) **(Fig. 6A, S3 Table)**. To investigate the mechanism by which MYBL2 overexpression facilitates epithelial-mesenchymal transition (EMT) in PCa, we conducted a screening of 1,184 EMT-related genes from the dbEMT 2.0 database. Notably, our findings indicated that the overexpression of MYBL2 predominantly influenced the expression of 12 key genes associated with epithelial-mesenchymal transition (EMT), with NOTCH3 emerging as the gene exhibiting the most significant differential expression in response** (Fig. 6B-C)**. QPCR assays also confirmed that MYBL2 overexpression promoted NOTCH3 gene expression **(Fig. 6D)**. The overexpression of MYBL2 facilitated the invasion and EMT of PCa cells, while NOTCH3 knockdown partly abrogated that **(Fig. 6E-F)**. In summary, MYBL2 overexpression facilitated PCa invasion and epithelial-mesenchymal transition (EMT) via increasing NOTCH3 expression.

### MYBL2 overexpression promotes the prostate cancer xenograft growth and bone metastasis in vivo

**Figure 7:**
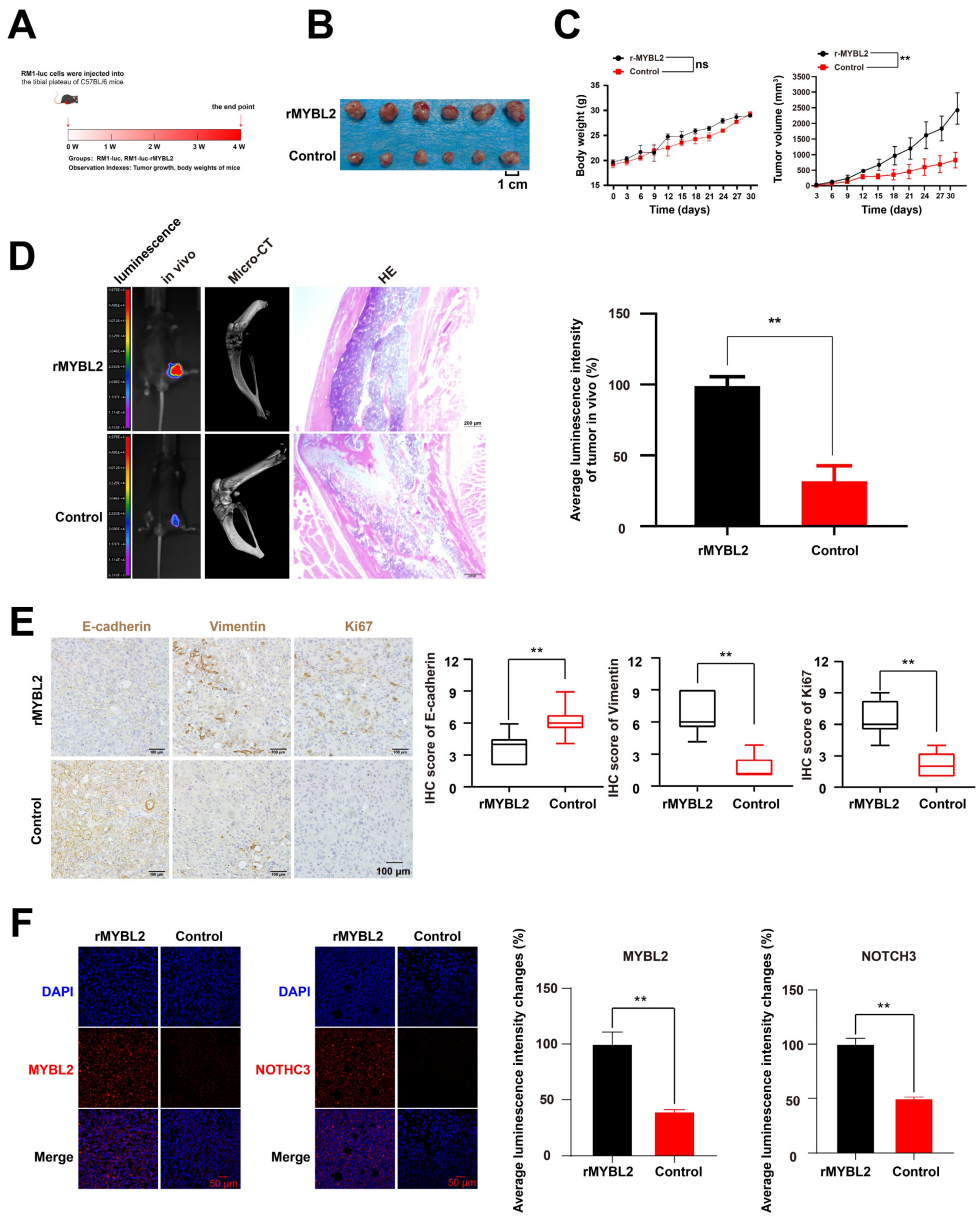
** MYBL2 overexpression promotes the PCa xenograft growth and bone metastasis in vivo. A**. Framework for the design of animal experiments. **B-D.** MYBL2 overexpression promoted the growth and bone metastasis of PCa xenograft. **E-F.** IHC assay and IF assay were used to detected the expression of target proteins. n = 3, **p < 0.01; ns represents no statistical significance.

In order to examine the impact of MYBL2 on tumor growth and metastasis in prostate cancer (PCa) in vivo, RM1-luc cells were administered into the tibial plateau region of C57BL/6 mice to establish a PCa xenograft metastasis model **(Fig. 7A)**. The findings demonstrated that MYBL2 overexpression not only facilitated the growth of PCa xenografts but also enhanced bone metastasis and exacerbated bone destruction associated with the xenograft tumors **(Fig. 7B-D)**. Furthermore, throughout the duration of the experiment, there were no fatalities among the mice, nor were there any significant alterations in body weight **(Fig. 7C)**. Examination of the transplanted tumor tissues revealed that the group with MYBL2 overexpression exhibited marked activation of epithelial-mesenchymal transition (EMT), as evidenced by an upregulation of Vimentin and a downregulation of E-cadherin expression **(Fig. 7E)**. Furthermore, MYBL2 overexpression was also induced the increased expression of Ki67 **(Fig. 7E)**. Immunofluorescence analysis demonstrated a significant upregulation of NOTCH3 expression in the tumor tissues of the group with MYBL2 overexpression **(Fig. 7F)**. In summary, the overexpression of MYBL2 facilitated the growth of PCa xenografts and promoted bone metastasis in murine models.

## Discussion

Prostate cancer (PCa) is the sixth most common cancer in 185 countries and the third leading cause of cancer-related deaths 1. Patients with early-stage PCa has a good prognosis and surgical resection is a common treatment strategy. As the disease advances to later stages, the availability of effective treatment strategies becomes significantly limited. A significant proportion of patients with PCa are diagnosed with distant metastasis at the time of initial presentation. In cases of advanced and metastatic PCa, the primary therapeutic approach is androgen deprivation therapy (ADT) 11. While ADT typically demonstrates short-term effectiveness, it ultimately results in the development of resistance and further metastatic progression 12. Consequently, it is imperative to conduct further investigations into the mechanisms underlying PCa metastasis.

Bioinformatics serves as a highly efficient and effective approach for integrating data from various studies. By aggregating such data, we can expedite the identification of key genes that influence poor prognosis and metastasis in PCa patients. For instance, utilizing a network vulnerability analysis model, a study demonstrated that five microRNAs including miR-204-5p, miR-198, miR-145-5p, miR-101-3p, and miR-152 were identified as potential biomarkers for predicting the metastasis of PCa 13. Building on this foundation, subsequent researchers can further investigate the mechanisms by which target microRNAs inhibit PCa metastasis. A study employing a range of molecular biology experiments and animal models in vivo elucidated that miR-204-5p can inhibit the invasion, migration, and bone metastasis of PCa cells through the inactivation of NF-κB signaling pathways 14. To identify the key genes responsible for PCa metastasis, three pertinent datasets including GSE3325, GSE27616, and GSE30994 were selected from the Gene Expression Omnibus (GEO) database. Through the development of a prognostic model and a cluster analysis conducted on a dataset comprising 498 patients with PCa from the TCGA database, MYBL2 was identified as a critical gene responsible for PCa metastasis.

The MYB proto-oncogene-like 2 (MYBL2) is a transcription factor within the MYB family, which plays a critical role in regulating the expression of a range of oncogenes 3. The MYB gene family includes three members—MYB, MYBL1, and MYBL2—that encode the transcription factors MYB, MYBL1, and MYBL2 4. Alterations in MYB have been documented in various cancers, and MYB family proteins have been implicated in tumor cell plasticity 15, therapy resistance 16, and metastasis 17. MYBL2 also plays an important role in the progression of PCa. A study indicated that high MYBL2 expression was positively related to advanced *TNM* stage, elevated PSA levels, and higher Gleason scores in patients with PCa 17. Notably, conventional androgen deprivation therapy (ADT) has been implicated in facilitating the metastatic progression of PCa 12. Approximately 70% of patients with metastatic prostate cancer exhibit bone metastases, which constitute the primary factor contributing to the unfavorable prognosis observed in these individuals 18-20 Furthermore, MYBL2 is intricately associated with the advancement of neuroendocrine prostate cancer (NEPC) 21, metastatic hormone-sensitive prostate cancer (mHSPC) 22, and the development of castration resistance 23. Metastasis plays a critical role in the poor prognosis observed in patients with prostate cancer (PCa) 2. Currently, patients with bone metastases resulting from prostate cancer continue to experience a limited availability of therapeutic options. The primary pharmacological interventions available to mitigate adverse bone-related events associated with bone metastases include bisphosphonates 24, denosumab 25, and a few other agents. A comprehensive understanding of the mechanisms underlying bone metastases in prostate cancer is crucial for the development of novel therapeutic agents. Therefore, this study aimed to elucidate the role and underlying mechanisms of MYBL2 in facilitating bone metastasis in prostate cancer. Our findings demonstrate that the overexpression of the MYBL2 molecule is significantly associated with the progression of bone metastasis in prostate cancer. These insights may inform the future development of targeted therapies against MYBL2 for the treatment of bone metastases in prostate cancer.

## Conclusions

This study provides robust evidence linking MYBL2 to poor prognosis in patients with prostate cancer (PCa), predominantly by promoting invasion and epithelial-mesenchymal transition (EMT). Mechanistic study elucidated that MYBL2 can facilitate the invasion and EMT of PCa cells via the increasing expression of NOTCH3. This finding established a foundational basis for exploring the MYBL2/NOTCH3 axis as a potential molecular target for therapeutic intervention in metastatic PCa.

## Supplementary Material

Supplementary tables.

## References

[1] Sung H, Ferlay J, Siegel RL, Laversanne M, Soerjomataram I, Jemal A (2021). Global Cancer Statistics 2020: GLOBOCAN Estimates of Incidence and Mortality Worldwide for 36 Cancers in 185 Countries. CA: a cancer journal for clinicians.

[2] Costello AJ (2020). Considering the role of radical prostatectomy in 21st century prostate cancer care. Nature reviews Urology.

[3] Musa J, Aynaud MM, Mirabeau O, Delattre O, Grünewald TG (2017). MYBL2 (B-Myb): a central regulator of cell proliferation, cell survival and differentiation involved in tumorigenesis. Cell Death Dis.

[4] Cicirò Y, Sala A (2021). MYB oncoproteins: emerging players and potential therapeutic targets in human cancer. Oncogenesis.

[5] Yoshikawa Y, Stopsack KH, Wang XV, Chen YH, Mazzu YZ, Burton F (2022). Increased MYBL2 expression in aggressive hormone-sensitive prostate cancer. Molecular oncology.

[6] Li Q, Wang M, Hu Y, Zhao E, Li J, Ren L (2021). MYBL2 disrupts the Hippo-YAP pathway and confers castration resistance and metastatic potential in prostate cancer. Theranostics.

[7] Jiao M, Zhang F, Teng W, Zhou C (2022). MYBL2 is a Novel Independent Prognostic Biomarker and Correlated with Immune Infiltrates in Prostate Cancer. International journal of general medicine.

[8] Bardou P, Mariette J, Escudié F, Djemiel C, Klopp C (2014). jvenn: an interactive Venn diagram viewer. BMC bioinformatics.

[9] Tang Z, Li C, Kang B, Gao G, Li C, Zhang Z (2017). GEPIA: a web server for cancer and normal gene expression profiling and interactive analyses. Nucleic acids research.

[10] Huang R, Wang S, Wang N, Zheng Y, Zhou J, Yang B (2020). CCL5 derived from tumor-associated macrophages promotes prostate cancer stem cells and metastasis via activating β-catenin/STAT3 signaling. Cell Death & Disease.

[11] Rebello RJ, Oing C, Knudsen KE, Loeb S, Johnson DC, Reiter RE (2021). Prostate cancer. Nature reviews Disease primers.

[12] Niu Y, Guo C, Wen S, Tian J, Luo J, Wang K (2018). ADT with antiandrogens in prostate cancer induces adverse effect of increasing resistance, neuroendocrine differentiation and tumor metastasis. Cancer letters.

[13] Lin Y, Chen F, Shen L, Tang X, Du C, Sun Z (2018). Biomarker microRNAs for prostate cancer metastasis: screened with a network vulnerability analysis model. Journal of translational medicine.

[14] Wa Q, Huang S, Pan J, Tang Y, He S, Fu X (2019). miR-204-5p Represses Bone Metastasis via Inactivating NF-κB Signaling in Prostate Cancer. Molecular therapy Nucleic acids.

[15] Anand S, Vikramdeo KS, Sudan SK, Sharma A, Acharya S, Khan MA (2024). From modulation of cellular plasticity to potentiation of therapeutic resistance: new and emerging roles of MYB transcription factors in human malignancies. Cancer metastasis reviews.

[16] Pan B, Wan T, Zhou Y, Huang S, Yuan L, Jiang Y (2023). The MYBL2-CCL2 axis promotes tumor progression and resistance to anti-PD-1 therapy in ovarian cancer by inducing immunosuppressive macrophages. Cancer cell international.

[17] Liu W, Shen D, Ju L, Zhang R, Du W, Jin W (2022). MYBL2 promotes proliferation and metastasis of bladder cancer through transactivation of CDCA3. Oncogene.

[18] Zhuyan J, Chen M, Zhu T, Bao X, Zhen T, Xing K (2020). Critical steps to tumor metastasis: alterations of tumor microenvironment and extracellular matrix in the formation of pre-metastatic and metastatic niche. Cell & bioscience.

[19] Dong B, Gu Y, Sun X, Wang X, Zhou Y, Rong Z (2024). Targeting TUBB3 Suppresses Anoikis Resistance and Bone Metastasis in Prostate Cancer. Advanced healthcare materials.

[20] Body JJ, Casimiro S, Costa L (2015). Targeting bone metastases in prostate cancer: improving clinical outcome. Nature reviews Urology.

[21] German B, Alaiwi SA, Ho KL, Nanda JS, Fonseca MA, Burkhart DL (2024). MYBL2 Drives Prostate Cancer Plasticity: Inhibiting Its Transcriptional Target CDK2 for RB1-Deficient Neuroendocrine Prostate Cancer. Cancer research communications.

[22] Huang Q, Liu M, Zhang D, Lin BB, Fu X, Zhang Z (2023). Nitazoxanide inhibits acetylated KLF5-induced bone metastasis by modulating KLF5 function in prostate cancer. BMC medi-cine.

[23] Li X, Jiao M, Hu J, Qi M, Zhang J, Zhao M (2020). miR-30a inhibits androgen-independent growth of prostate cancer via targeting MYBL2, FOXD1, and SOX4. The Prostate.

[24] Vale CL, Burdett S, Rydzewska LHM, Albiges L, Clarke NW, Fisher D (2016). Addition of docetaxel or bisphosphonates to standard of care in men with localised or metastatic, hormone-sensitive prostate cancer: a systematic review and meta-analyses of aggregate data. The Lancet Oncology.

[25] Smith MR, Saad F, Oudard S, Shore N, Fizazi K, Sieber P (2013). Denosumab and bone metastasis-free survival in men with nonmetastatic castration-resistant prostate cancer: exploratory analyses by baseline prostate-specific antigen doubling time. Journal of clinical oncology: official journal of the American Society of Clinical Oncology.

